# Factors affecting the quality of working life among nurses caring for Syrian refugee camps in Jordan

**DOI:** 10.1186/s12960-023-00884-8

**Published:** 2024-01-02

**Authors:** Islam Oweidat, Abeer Omari, Mohammed ALBashtawy, Al Omar Saleh, Tahani Alrahbeni, Khalid Al-Mugheed, Ayman Daifallah Ismail Alsheikh

**Affiliations:** 1 Community and Mental Health Nursing Department, Zarqa University, Zarqa, Jordan; 2 Princess Salma Faculty of Nursing, Al-Bayt University, Mafraq, Jordan; 3 Clinical Nursing Department, Zarqa University, Zarqa, Jordan; 4 Molecular Toxicology and Genetics/College of Nursing, Riyadh Elm University, Riyadh, Saudi Arabia; 5 College of Nursing, Riyadh Elm University, Riyadh, Saudi Arabia; 6 Faculty of Allied Medical Sciences, Department of Medical Laboratory Science, Zarqa University, Zarqa, Jordan

**Keywords:** Quality of work life, Nurses, Refugee camps, Syrian refugees, Jordan, Healthcare, Humanitarian crisis

## Abstract

**Objectives:**

The global refugee crisis, exacerbated by the Syrian war, has placed tremendous strain on Jordan’s healthcare system and infrastructure, notably impacting nurses working in refugee camps. The aim to identify factors influencing nurses’ Quality of life at work (QWL) and understand their significance in crisis healthcare environments.

**Methodology:**

A cross-sectional study was conducted in multiple healthcare facilities within Syrian refugee camps. A convenient sample of 166 nurses participated, and data were collected using the Brook’s Quality of Nursing Work Life Survey. Data analysis included descriptive and inferential (one-way ANOVA) statistics. Significance level was set at 0.05.

**Results:**

Nurses in this study generally reported a moderate QWL, with an average score of 152.85, indicating that their overall work experience falls into the moderate range. The study found that nurses perceived their work-life/home-life balance (mean score 25.79), work design (mean score 35.71), work context (mean score 71.37), and work world (mean score 19.96) at levels indicative of moderate satisfaction. There were no statistically significant differences in QWL among participating nurses, suggesting that factors other than demographic characteristics may play a more influential role in determining nurses' QWL in the unique context of refugee caregiving.

**Conclusion:**

This study underscores that working within refugee healthcare missions and recommends targeted interventions to enhance their well-being.

## Background

The worldwide problem of refugees seeking safety and shelter in response to countries plagued by violence has become prominent [1]. The Syrian war, which began in 2011, has resulted in a significant humanitarian crisis of considerable magnitude in the twenty-first century. This crisis has caused the displacement of millions of Syrians from their residences, compelling them to seek asylum in neighboring nations [2]. Being close to Syria, Jordan has played a prominent role in accommodating a significant population of Syrian refugees. The recent surge in population has not only exerted considerable pressure on Jordan’s healthcare but has also presented notable obstacles to the healthcare system inside the country [3]. In the intricate context described, healthcare professionals, namely nurses, have assumed a crucial role in delivering treatment and assistance to Syrian refugees who are now staying in camps located inside the borders of Jordan [4].

The Quality of Working Life (QWL) concept has gained increasing attention in recent years, particularly in healthcare professions, explicitly focusing on nurses [5]. The QWL comprises several aspects, including job satisfaction, work-related stress, workload, organizational support, and the general well-being of healthcare employees [6]. In the context of nurses working in settings marked by humanitarian crises and healthcare delivery to marginalized people, comprehending the determinants that impact their QWL is not just a question of professional significance but also an ethical obligation [7].

It is essential to acknowledge the intricate and challenging circumstances under which healthcare personnel function inside Syrian refugee camps [4]. The healthcare worker above encounter various problems that substantially influence the quality of their professional experiences and their capacity to provide efficient treatment [7]. The challenges faced by healthcare professionals in emergency and critical care settings include factors such as a significant influx of patients, scarcity of resources [3], regular exposure to traumatic and distressing circumstances [7, 8], difficulties arising from language and cultural differences [9], and the demanding nature of emergency and critical care services [2]. Furthermore, the situation surrounding these refugee camps is further intricate due to resource limitations, overcrowding [10], and the immense scale of the catastrophe [11]. The comprehension of the elements that impact the QWL of nurses within this setting extends beyond a simple professional consideration; it assumes a position of ethical and humanitarian importance [1].

The well-being of healthcare providers is intricately linked to the quality of care they deliver. Studies consistently show that satisfied and motivated healthcare professionals provide higher-quality care [1]. In refugee camps, the quality of care is paramount, as refugees often arrive with complex physical and mental health needs resulting from trauma and displacement [2]. By enhancing the QWL of nurses, we indirectly contribute to improved healthcare outcomes for refugees [3].

High staff turnover rates, burnout, and job dissatisfaction can disrupt the continuity of care, which is especially critical in healthcare settings dealing with vulnerable populations [1–3]. This disruption can lead to fragmented healthcare services and increased patient vulnerability [5, 7]. Investigating and addressing factors affecting QWL can help satisfaction and staff turnover and promote the continuity of care [12, 13].

### Aim of the study

The primary aim of this study was to assess the quality of working life among nurses stationed in Jordanian refugee camps. Specifically, it sought to investigate the relationship between the quality of work life among nurses responsible for caring for refugees in emergency and medical care departments and various socio-demographic variables.

### Research questions

1. What are the levels of quality of working life among nurses working with Syrian refugees in Jordanian Camps?
2. Are there statistically significant differences in the Quality of Work Life (QWL) among nurses serving as caregivers for refugees in emergency and medical care departments based on their (gender, age, type of institution, marital status, education level, job designation, and number of years of experience)?

## Methodology

### Design

A descriptive, cross-sectional design was used in the current study.

### Settings and sampling

The study was conducted at various healthcare facilities within Syrian refugee camps in Jordan. These facilities included ten healthcare centers which deliver healthcare services for Syrian refugees at the east of Jordan. Convenience sampling was employed in this study to select the research participants. This method was chosen primarily due to its practicality and accessibility. Given the challenging and often resource-constrained setting of Syrian refugee camps, obtaining a complete list or conducting a random sampling of nurses could be logistically challenging and time-consuming. Convenience sampling allowed for a more efficient approach by including all available nurses in these camps.

The inclusion criteria for this study were intentionally broad to ensure a diverse and representative sample of nurses. Regardless of their roles, departments, specializations, years of study, academic certificates, work experience, and nationality, all registered nurses were eligible to participate. This inclusivity aimed to capture the full spectrum of experiences and perspectives within the nursing workforce in Syrian refugee camps.

Based on an estimated effect size of (*d*) = 0.7, *ά* = 0.05, power = 0.95, the required sample size was estimated at 138 to run a paired sample t-test. However, to account for potential attrition and the possibility of dropped questionnaires during data collection, an additional 20 percent of the calculated sample size was added. Therefore, a final sample size of 186 nurses was deemed sufficient to achieve the desired statistical power and to ensure that the study’s objectives could be met effectively.

### Measurement

In this study, several socio-demographic and work-related variables were measured to understand the characteristics of the registered nurses working in Syrian refugee camps in Jordan. Measuring these variables was crucial for assessing the factors influencing this population’s quality of work life (Gender, age, type of healthcare center, marital status, educational level, job designation, number of years of experience).

#### Brook’s quality of nursing work life survey (BQNWLS)

The study used BQNWLS scale which is a 42-item created to measure nurses’ work-life quality [14]. Each question enables respondents to rate their level of agreement or disagreement on a six-point scale ranging from 1 (strongly disagree) to 6 (strongly agree). The overall score for the BQNWLS is calculated by summing the values of all 42 questions, and it spans from 42 to 252, with a higher number reflecting superior QWL. The scale’s cut points for the total score were established as follows to reflect the degrees of QNWL: low (42–112), moderate (113–182), and high (183–252) [15]. The 42 questions are integrated to generate four components: work life/home life dimension (seven items, score range 7–42), representing the interaction between nurses’ jobs and home life. This component also represents the nurses’ roles in taking care of children (mother role), aging parents (daughter role), and family members (spouse role). The work design dimension (10 items with a score range of 10–60) depicts the actual job done by nurses. As a result, it assesses the nurses’ actual work environment, including workload, staffing, and independence. The work context dimension (20 items, score range of 20–120) represents the available resources to nurses in the clinical setting, such as continuous learning and the influence of the workplace environment on the patient and the nurse. Moreover, this component investigates the interaction between managers, employees, and other healthcare team members. Lastly, the work world dimension (5 questions, range of scores is 5–30) shows the impact of change and societal pressures on nursing. This factor is linked with nurses’ social standing and employment stability. The internal consistency coefficient of the BQNWL was (Cronbach = 0.89). Brooks et al., observed test–retest solid reliability for the overall BQNWL scores among 53 registered nurses across 14 days between testing (*r* = 0.90, *P* 0.001) [14]. Almalki et al., translated the measurement tool in Arabic and applied to an Arabic nursing population (*n* = 508) in which the internal consistency reliabilities of the total BQNWL scores were (0.89) [15]; consequently, this Arabic version of the survey was in the current study.

### Data collection

The researcher-initiated collaboration with the Chief Nurse Officer (CNO) at each hospital within the Syrian refugee camps. These meetings were conducted in the first two weeks of April 2023. During these sessions, the CNOs were provided with a thorough explanation of the study's objectives, data collection procedures, and logistical requirements. The questionnaire did not request their names or identifying information to safeguard participants’ privacy and confidentiality. Respondents were assured that their responses would be treated with the utmost confidentiality and would not be linked back to them in any way.

Data collected during the study were securely stored on the investigator’s personal computer, which was password-protected to prevent unauthorized access. Additionally, participants were encouraged to reach out with any questions or for clarification, and they were provided with the researcher’s email contact for this purpose. Throughout all stages of the study, ethical guidelines established by the institutional research committee at Zarqa University were strictly adhered to. Furthermore, the research was conducted by the principles outlined in the 1964 Helsinki Declaration and its subsequent revisions and any other applicable ethical standards.

### Data analysis

With the dataset prepared, descriptive statistical analyses were conducted to gain insights into the variables’ central tendencies, distributions, and variabilities under investigation. These descriptive statistics helped provide a clear and concise summary of the critical socio-demographic and work-related characteristics of the nursing workforce in Syrian refugee camps in Jordan. To investigate the relationships between key variables were tested by ANOVA. The significance was set at less than 0.05.

### Ethical considerations

Written permission was sought from the authors [15] to utilize the scales in this study. Ethical approval was obtained from the Institutional Review Board (IRB) of Zarqa University (5/2022 IRB at Zarqa University), specifically from the Faculty of Nursing. Subsequently, a formal cover letter issued by Zarqa University was submitted to the Ministry of Health to secure IRB approval for the study. Participants were assured that their participation was entirely voluntary, with no compulsion. They were explicitly informed of their right to withdraw from the study at any point without facing any negative consequences or repercussions.

## Results

### Description of the study population

The participants’ ages were distributed, with the majority in the 31–40 age range (58.6%), and followed by 21–30 aged (21.0%). In terms of gender distribution, the majority of participants were male (57.5%). Most participants were affiliated with healthcare centers (91.9%) in terms of insurance type. The majority of participants were married (73.7%), educational qualifications varied among the participants, with the highest proportion holding a Bachelor’s degree (60.2%), regarding job designations, most participants identified themselves as registered nurse (57.5%). The majority of participants were had 6–10 years of experience (41.9%). Table 1

**Table 1:** Demographic characteristics of participants (*N* = 186)

Variable	Category	Frequency	Percent
Age	21–30	39	21.0
	31–40	109	58.6
	41–50	35	18.8
	51–55	3	1.6
Gender	Male	107	57.5
	Female	79	42.5
Insurance type	Tertiary	4	2.2
	Local	7	3.8
	Specialized clinics	4	2.2
	Healthcare center	171	91.9
Marital status	Single	44	23.7
	Married	137	73.7
	Widowed	1	0.5
	Divorced	4	2.2
Educational level	Diploma	42	22.6
	Post diploma	26	14.0
	Bachelor	112	60.2
	Masters	6	3.2
Job designation	General practicing nurse	107	57.5
	Charge nurse	62	33.3
	Head nurse	13	7.0
	Nursing supervisor	4	2.2
Years of experience	1–5	49	26.3
	6–10	78	41.9
	11–15	49	26.3
	16–20	2	1.1
	21–25	5	2.7
	25 or more	3	1.6

The mean score for the QWL total was 152.85, with a standard deviation of 27.52. The highest mean was QWL work-context subscale (*M* = 71.3 ± 15.2), followed by QWL work-design subscale (*M* = 35.7 ± 7.4). The lowest mean subscale was QWL work-world (*M* = 19.9 ± 4.12) Table 2.

**Table 2:** Descriptive statistics for QWL total and subscale scores

	*N*	Minimum	Maximum	Mean	Std. deviation
Total QWL score	186	87.00	210.00	152.85	27.52
QWL work-life and home-life subscale	186	12.00	35.00	25.79	4.70
QWL work-design subscale	186	20.00	50.00	35.71	7.49
QWL work-context subscale	186	31.00	100.00	71.37	15.28
QWL work-world subscale	186	10.00	25.00	19.96	4.12

The mean QWL score for male nurses was 153.35 (SD = 28.79), while female nurses had a mean score of 152.17 (SD = 25.87). The independent t-test showed a non-significant difference between the two groups (*t* = 0.292, *p* = 0.770). Consequently, the findings did not show any statistically significant variations in QWL scores based on gender among these nurses, suggesting that gender is not a significant determinant of QWL in this context (Table 3).

**Table 3:** Variations in total QWL score based on gender (*N* = 186)

Variable	Category	*N*	Mean	Std. deviation	*T*	Sig
Gender	Male	107	153.35	28.79	0.292	0.770
	Female	79	152.17	25.87		

There were no statistically significant differences obtained between age and insurance type. Marital status also did not yield statistically significant differences in QWL scores (*F* = 1.53, *p* = 0.21). Likewise, educational levels (Diploma, Post Diploma, Bachelor, Master) and job designations (general practicing nurse, charge nurse, head nurse, nursing supervisor) did not result in significant variations in QWL scores (*F* = 0.85, *p* = 0.46 for education, and *F* = 0.12, *p* = 0.94 for job designation). Finally, years of experience (ranging from 1–5 to 25 or more years) showed no statistically significant impact on QWL scores (*F* = 0.51, *p* = 0.76). Table 4.

**Table 4:** Variations in total QWL score based on demographic characteristics of more than two expressions (*N* = 186)

Variable	Category	*N*	Mean	Std. deviation	*F*	Sig
Age	21–30	39	153.25	24.13	0.27	0.84
	31–40	109	152.28	28.68		
	41–50	35	155.17	28.37		
	51–55	3	141.33	24.44		
Insurance type	Tertiary	4	143.00	17.64	0.96	0.41
	Local	7	137.42	7.06		
	Polyclinic	4	152.00	40.38		
	Healthcare center	171	153.73	27.85		
Marital status	Single	44	149.04	23.45	1.53	0.21
	Married	137	154.62	28.05		
	Widowed	1	106.00	4.09		
	Divorced	4	146.00	44.78		
Educational level	Diploma	42	157.71	27.35	0.85	0.46
	Post diploma	26	153.34	26.83		
	Bachelor	112	150.51	27.71		
	Masters	6	160.33	29.37		
Job designation	General practicing nurse	107	153.73	28.34	0.12	0.94
	Charge nurse	62	152.14	27.52		
	Head nurse	13	149.38	23.44		
	Nursing supervisor	4	151.50	25.52		
Years of experience	1–5	49	151.81	25.66	0.51	0.76
	6–10	78	154.11	29.08		
	11–15	49	152.71	27.40		
	16–20	2	166.00	7.07		
	21–25	5	152.60	36.03		
	25 or more	3	131.00	9.53		

## Discussion

The fundamental objective of this study was to delve into the intricacies of QWL among nurses serving in emergency and medical care departments within the context of providing healthcare to Syrian refugees in Jordanian camps. By doing so, the study sought to contribute meaningful insights into the work experiences of these dedicated healthcare professionals, ultimately advancing our comprehension of the challenges and dynamics associated with such a noble yet demanding profession.

The study revealed that the levels of QWL were moderate among nurses. The results align with previous study that focused demanding nature of nursing such as quality of work life in humanitarian and crisis settings [16]. The comparative study involving Thai and Japanese primary health nurses unraveled a modest level of QWL for both groups [17]. In Bangladesh, a study found that nurses rated their QWL as moderate [18]. In contrast, an Iranian study demonstrated that 69.3% of nurses expressed dissatisfaction and reported a poor QWL [19]. Similarly, a study among ICU nurses in Gaza Strip/Palestine found moderate levels of QWL [20]. The moderate level of satisfaction indicates a careful balance between the rewarding aspects of caregiving and the huge challenges inherent to working in such a high-pressure environment. The moderate rating of this subscale underscores the need for support mechanisms and interventions that promote better work-life integration for nurses in these circumstances [21]. Interventions suggested to enhance QWL resonate with the notion that QWL can be improved through various strategies, including teamwork enhancement and stress management programs. This implies that nursing professionals can benefit from tailored interventions to augment their QWL.

Biresaw and colleagues’ study among Ethiopian nurses found that 40.8% reported an excellent QWL [22]. Another study among Indian nurses revealed that most reported a moderate QWL level, with some reporting an excellent QWL [23]. Although the context is different from that in Jordan, this finding serves as a reminder that favorable QWL outcomes are attainable within nursing professions, even in resource-constrained environments.

Factors such as salary, job stress, and involvement in decision-making influenced QWL, signifying the intricate dynamics nurses navigate within their work environments. This study underscores that suboptimal QWL is not exclusive to resource-limited settings but can manifest across diverse healthcare contexts. In Saudi Arabia, nurses were found to be dissatisfied with their work lives [15]. Factors such as working hours, family–work balance, and professional development opportunities underscore the multifaceted nature of QWL within primary healthcare settings. The satisfaction with co-workers and a sense of belonging hints at the significance of interpersonal relationships in mitigating the impact of challenging work conditions.

Collectively, the literature on QWL among nurses in different settings outside refugee camps provides valuable context. While some studies found moderate-to-excellent levels of QWL [18, 20–22], others revealed unsatisfactory QWL levels [16, 24]. These variations underscore the importance of understanding the contextual factors influencing QWL, which might differ considerably between settings.

Age, often regarded as a pivotal determinant in career satisfaction [25, 26], did not significantly impact nurses' QWL in our study. This surprising result indicates that, within the demanding environment of refugee camps, nurses of varying age groups experienced comparable levels of satisfaction and contentment in their work roles. This contradicts some previous research but resonates with the notion that humanitarian contexts can create unique dynamics [27–29], potentially overshadowing age-related differences in QWL.

The findings also indicated that insurance type, marital status, education level, job designation, and years of experience did not lead to significant differences in QWL among nurses in the refugee camp context. These results suggest that the overriding mission of providing critical care to a vulnerable population may act as a great equalizer, minimizing distinctions that might typically influence QWL in conventional nursing settings. In contrast to our findings, Jaber et al. highlighted the positive correlation between the quality of work life and organizational commitment, emphasizing the importance of measures such as teamwork and competitive salaries [30]. While this correlation may hold in other healthcare settings, our study suggests that within the unique context of refugee camp nursing, the pressing humanitarian needs could eclipse the influence of organizational factors.

In interpreting the current study’s findings regarding the factors affecting QWL among nurses caring for Syrian refugees in Jordanian camps, it is evident that the absence of statistically significant factors can be attributed to the unique and challenging context of refugee camp nursing. While it might initially appear surprising that factors such as gender, age, insurance type, marital status, education level, job designation, and years of experience did not significantly impact nurses’ QWL, a closer examination provides insights into why this might be the case.

One plausible explanation is the overriding mission and circumstances of nursing in a humanitarian setting [31]. The primary objective for nurses in refugee camps is to provide critical healthcare to a vulnerable population enduring challenging conditions [32, 33]. This overarching goal can overshadow the influence of conventional demographic factors. In such contexts, the nurses' dedication to their mission and the immediate needs of the refugee population might be the predominant drivers of their job satisfaction and QWL. Therefore, factors that typically affect QWL in more conventional healthcare settings, such as organizational policies, job titles, or individual characteristics, may not hold the same weight.

Additionally, the pressing nature of the humanitarian mission in refugee camps could create a sense of camaraderie and shared purpose among nurses [34], transcending the usual distinctions that might lead to significant variations in QWL. In this environment, nurses may find motivation and job satisfaction in the knowledge that they are making a profound difference in the lives of refugees, regardless of their individual characteristics or organizational factors [35].

Moreover, it is essential to consider the potential homogeneity of the nursing workforce in this context. Nurses working in refugee camps may share similar values, motivations, and levels of commitment due to their deliberate choice to work in such challenging environments [4]. This shared dedication might minimize the variability in QWL that can arise from demographic or organizational differences in more diverse healthcare settings.

Furthermore, the absence of significant factors influencing QWL in this study might suggest different priorities for refugee camp nurses. Rather than seeking job satisfaction through factors like career advancement or work-life balance, they might derive their sense of fulfillment from the tangible impact they make on the lives of refugees. The immediacy of the humanitarian need can reshape their professional values and what they perceive as necessary for their QWL [35].

In speculation, these findings prompt us to reflect on the nature of nursing in humanitarian settings and its implications for healthcare systems. The absence of significant factors impacting QWL may reflect nurses' unique commitment and resilience in such challenging environments. However, it also raises questions about the sustainability of this model. While the unwavering dedication of nurses to their mission is admirable, it may not be a substitute for comprehensive support systems and policies that address their well-being.

### Limitations

This study has several limitations, including its cross-sectional design, reliance on self-reported data susceptible to response bias, limited generalizability to other healthcare contexts, potential recall bias, language, and cultural factors, the scope of variables considered, sole reliance on nurses' perspectives, and the evolving nature of the refugee camp healthcare context. Acknowledging these limitations is crucial for interpreting the findings accurately and guiding future research efforts toward understanding the QWL of nurses in Syrian refugee camps in Jordan.

## Conclusion

In conclusion, this study provides critical insights into the QWL among nurses caring for Syrian refugees in Jordanian camps. It reveals that nurses generally reported moderate QWL levels, with no significant differences based on demographic factors. It also has practical implications, emphasizing the need for targeted interventions to enhance nurses’ well-being in refugee camp settings. Overall, this study advances our understanding of nurses’ experiences in refugee care and underscores the importance of supporting their QWL to improve healthcare delivery for vulnerable populations.

## Data Availability

The data that support the findings of this study are available on request from the corresponding author.
